# Inflammatory activity affects the accuracy of liver stiffness measurement by transient elastography but not by two‐dimensional shear wave elastography in non‐alcoholic fatty liver disease

**DOI:** 10.1111/liv.15116

**Published:** 2021-12-03

**Authors:** Yuly P. Mendoza, Susana G. Rodrigues, Maria G. Delgado, Giuseppe Murgia, Naomi F. Lange, Jonas Schropp, Matteo Montani, Jean‐François Dufour, Annalisa Berzigotti

**Affiliations:** ^1^ Department of Visceral Surgery and Medicine Inselspital‐ Bern University Hospital University of Bern Bern Switzerland; ^2^ Graduate School for Health Sciences (GHS) University of Bern Bern Switzerland; ^3^ Department of Computer Science Open University of Cyprus Cyprus; ^4^ Department of Psychology University of Cyprus Nicosia Cyprus; ^5^ Institute of Pathology Inselspital Bern University Hospital University of Bern Bern Switzerland

**Keywords:** Fibroscan, fibrosis, liver stiffness measurement, NASH, non‐invasive test

## Abstract

**Background:**

In patients with non‐alcoholic fatty liver disease (NAFLD), the impact of the severity of steatosis and inflammatory activity on the accuracy of liver stiffness measurement (LSM) by transient elastography (TE) and by two‐dimensional shear wave elastography (2D‐SWE) in staging liver fibrosis is still debated and scarce. We aimed to focus on this aspect.

**Methods:**

We prospectively studied 104 patients requiring biopsy for the assessment of NAFLD. We used ordinary least squares regression to test for differences in the association between fibrosis and LSM by TE and 2D‐SWE when other factors (steatosis and inflammatory activity) are considered.

**Results:**

Among 104 patients, 102 had reliable LSM by TE, and 88 had valid LSM by 2D‐SWE. The association between fibrosis based on histology and LSM was significantly stronger when 2D‐SWE assessed LSM compared to TE (Spearman's correlation coefficient of .71; *P* < .001 vs .51, *P* < .001; *Z* = 2.21, *P* = .027). Inflammatory activity was an independent predictor of LSM by TE but not of LSM by 2D‐SWE. After controlling for fibrosis, age, sex and body mass index, the inflammatory activity and the interaction between inflammatory activity and fibrosis independently explained 11% and 13% of variance in LSM by TE respectively. Steatosis did not affect the association of fibrosis and LSM by either method.

**Conclusion:**

Inflammatory activity on histology significantly affects LSM by TE, but not LSM by 2D‐SWE in NAFLD. LSM by 2D‐SWE reflects liver fibrosis more accurately than LSM by TE. Furthermore, the severity of steatosis on histology did not influence the association of LSM and fibrosis by either elastography method.

**Abbreviations**
2D‐SWE: two‐dimensional shear wave elastography
ALT: alanine aminotransferase
BMI: body mass index
GGT: gamma‐glutamyl transpeptidase
LSM: liver stiffness measurement
NAFLD: non‐alcoholic fatty liver disease
NASH: non‐alcoholic steatohepatitis
TE: transient elastography

**Key points**

In patients with non‐alcoholic fatty liver disease (NAFLD), the impact of steatosis and inflammatory activity on the accuracy of liver stiffness measurement is still debated. Therefore, we compared two non‐invasive tests, transient elastography (Fibroscan^®^) and two‐dimensional shear wave elastography (2D‐SWE), in this population. We found that steatosis on histology did not affect liver stiffness by Fibroscan and 2D‐SWE. Whereas the inflammatory activity significantly affects liver stiffness by Fibroscan but not by 2D‐SWE. Furthermore, liver stiffness by 2D‐SWE was better correlated with fibrosis stage. Thus, liver stiffness using 2D‐SWE appears to reflect liver fibrosis more accurately than Fibroscan in this population of NAFLD patients.

## 1 INTRODUCTION

Liver fibrosis is the most important prognostic factor in patients with non‐alcoholic fatty liver disease (NAFLD).^1^ Since NAFLD is rapidly increasing and is already the most common cause of chronic liver disease worldwide^2^, ^3^ and one of the main indications for liver transplantation,^4^, ^5^ reliable methods to stage liver fibrosis in NAFLD are an urgent need in clinical practice. Liver biopsy remains the reference standard to characterize non‐alcoholic steatohepatitis (NASH).^6^ Nevertheless, liver biopsy is an invasive and expensive procedure associated with patient discomfort and sampling variability.^7^, ^8^ Elastography techniques, such as transient elastography (TE) and two‐dimensional shear wave elastography (2D‐SWE), provide a physical measure of liver stiffness which is closely related to fibrosis in chronic liver disease,^9^ and have emerged as an alternative to liver biopsy.

Although TE has been validated in NAFLD as a method to identify and stage fibrosis, it has been shown that steatosis and inflammatory activity might influence the accuracy of liver stiffness measurements (LSM) to predict fibrosis.^9^ However, the data regarding the impact of steatosis on LSM are still controversial. Some studies show no association,^10^, ^11^, ^12^ one study showed that severe steatosis leads to an overestimation of liver fibrosis by LSM assessed using TE,^13^ and another one showed that steatosis leads to an underestimation of liver fibrosis by LSM assessed by TE.^14^ To date, only one study evaluated the influence of steatosis and inflammation on LSM using 2D‐SWE in NAFLD,^15^ and no head‐to‐head study compared TE and 2D‐SWE vs histology with the aim of addressing which of the two methods is mostly influenced by confounders (steatosis and inflammation).

This study aimed to assess whether histological steatosis and inflammatory activity in NAFLD patients affects the accuracy of LSM by two different ultrasound elastography methods (TE and 2D‐SWE) in predicting fibrosis.

## 2 PATIENTS AND METHODS

### 2.1 Study population

We prospectively included consecutive adult patients with NAFLD who underwent liver biopsy from August 2018 through September 2020 who had LSM performed using TE and 2D‐SWE within 2 months from liver biopsy at an academic tertiary centre. Exclusion criteria were as follows: liver disease of other aetiology (chronic hepatitis B or C, autoimmune hepatitis, primary biliary cholangitis, primary sclerosing cholangitis, genetic hemochromatosis, drug‐induced hepatotoxicity, a‐1 antitrypsin deficiency, Wilson's disease, etc); exposure to drugs that can cause secondary NAFLD (corticosteroids, amiodarone and tamoxifen); significant alcohol consumption (>3 standard drinks/day in men and >2 drinks/day in women, or binge drinking defined as >5 standard drinks in men and >4 in women over a 2‐hour period)^3^; ALT >5 times the upper limit of normality; and refusal of consent to further use of personal health‐related data for research.

The study was performed according to the principles of the Declaration of Helsinki, and approval was obtained from the local ethics committee (KEK BE 2018‐00487).

### 2.2 Clinical and laboratory assessment

Demographic, clinical, anthropometric and laboratory data were collected at the time of the biopsy. Obesity was defined as body mass index (BMI) ≥30 kg/m^2^. Diabetes mellitus was defined as a fasting glycaemia ≥7 mmol/L or an HbA1c ≥6.5%, or current antidiabetic treatment. The presence of arterial hypertension and dyslipidaemia was recorded from the clinical charts.

### 2.3 LSM by transient elastography (TE)

Transient elastography (TE 502 Touch; Echosens, Paris, France) provided with M and XL probe was used to assess LSM, following the EASL‐ALEH clinical practice guidelines (M probe in patients with BMI <30 kg/m^2^ and XL probe in obese patients and/or skin‐to‐capsule distance ≥25 mm). Measurements were performed in a fasting state during a routine visit at our hepatology outpatient facility. Only patients with 10 valid measurements were included in the study. IQR/M values ≥.30 were considered unreliable; no valid shots were considered as failures of the technique.

### 2.4 LSM by two‐dimensional shear wave elastography

LSM by 2D‐SWE was performed using the Aixplorer ultrasound system (SuperSonic Imagine SA, Aix‐en‐Provence, France). Patients were placed in a supine position, with the right arm in extension. The operator selected a region of the right lobe of the liver with good spatial resolution for B‐mode ultrasound imaging, free of large vascular structures and at least 15 mm below the capsule through a right intercostal space, and during breath hold activated 2D‐SWE. Once a colour map with complete and homogeneous filling was obtained in the assessment area, a region of interest 15 mm in diameter was positioned in the centre of the colour map to measure stiffness using the Q box tool. We obtained three successful and valid measurements for each patient and used the mean value and the standard deviation of these measurements as liver stiffness measure.^16^ Variability (SD) over 30% of the average liver stiffness value was considered as unreliable measurement.^17^ When the operator obtained little or no signal in the region of interest for all acquisitions, the measurements were defined as failures.

### 2.5 Histological assessment

Liver biopsies were assessed by one experienced pathologist. NAFLD severity was scored according to the SAF scoring system^6^ that evaluates individually the grade of steatosis, activity and fibrosis. The steatosis grade was classified by the percentage of hepatocytes containing large‐ and medium‐sized intracytoplasmic lipid droplets, on a scale of 0‐3 (0: <5%; 1: 5%‐33%; 2: 34%‐66%; and 3: >67%). The grade of inflammatory activity was rated from A0 to A4 by addition of grades of ballooning and lobular inflammation, each graded from 0 to 2. Ballooning of hepatocytes was defined as the presence of hepatocyte clusters with a round shaped and pale cytoplasm (0: normal hepatocytes; 1: ballooning but normal size; and 2: ballooning with at least one enlarged ballooned hepatocyte). Lobular inflammation was defined as a focus of two or more inflammatory cells within the lobule organized either as microgranulomas or located within the sinusoids. Foci were counted at 20× magnification (grade 0: none; 1: ≤2 foci per lobule; and 2: >2 foci per lobule). The stage of fibrosis (F, from F0 to F4), was assessed according to the NASH Clinical Research Network staging system, with the single modification of pooling the three substages (1a, 1b and 1c) into a single F1 score. The diagnosis of NASH had >5% steatosis in hepatocytes and a grade of activity A ≥ 2.^6^, ^18^ Moreover, the NAFLD activity score (NAS) was calculated. NAS is the unweighted sum of steatosis grade, lobular inflammation and ballooning, ranging from 0 to 8 according to the grades of steatosis (0‐3), lobular inflammation (0‐3) and hepatocellular ballooning (0‐2).^19^ Interpretability for liver biopsy was based on the standard criteria of length, number of portal tracts (>10) and lack of major fragmentation.

### 2.6 Statistical analysis

Continuous variables are described as mean ± standard deviation, categorical variables as a number of cases (percentage). These descriptive statistics are provided for the complete group (104 patients) and the subgroups with valid LSM by TE and by 2D‐SWE. The subgroups of patients with and without valid SWE were compared using Welch's *t*‐test where parametric assumptions are adequate. For ordinal variables, Wilcoxon's rank‐sum test was used. Categorical variables were compared using the chi‐squared test. Missing values on all predictor variables were handled using Multiple Imputation by Chained Equations with *M* = 20 imputations. The two non‐invasive tests, LSM by TE and 2D‐SWE, were not used as predictors when creating imputations for other variables and were not imputed themselves. Bivariate associations between patient demographic characteristics, laboratory and liver histology parameter, and the two non‐invasive tests, LSM by TE and LSM by 2D‐SWE, were calculated using pairwise Spearman's correlations on multiply imputed data.

We performed a model comparison against the baseline model with fibrosis stage on Natural log (ln) LSM by TE and ln LSM by 2D‐SWE. Covariates as well as their interactions with fibrosis stage were added to the baseline model one‐by‐one. The extended models were compared to the baseline models using an *F*‐test to determine whether the addition of the covariate would add explained variance to the baseline model. All model comparisons were adjusted for multiple testing using the method by Holm. Ordinal variables were coded as treatment contrasts. Possible main effects or interactions of the covariate were then only further examined if the model explained significantly more variance than the baseline model. In addition, one more complex model was calculated for LSM by TE, which included patient characteristics as control variables as well as all significant covariates from the prior analyses. All reported *P*‐values are two‐sided and values <.05 were considered statistically significant. All confidence intervals are at the 95% level. Statistical analyses were conducted using R open‐source statistical software, version 4.0.3.

## 3 RESULTS

### 3.1 Characteristics of the study population

One hundred and four patients undergoing liver biopsy to grade and stage NAFLD were evaluated using liver histology, relevant laboratory parameters and LSM by TE and 2D‐SWE. One hundred and two had reliable LSM by TE and 88 had reliable LSM by 2D‐SWE. The differences between the 88 patients with reliable LSM by 2D‐SWE and the 16 patients without reliable LSM by 2D‐SWE are shown in Table S1. Eighty‐six patients had reliable LSM using both TE and 2D‐SWE methods; a flowchart of patient inclusion and exclusion is provided in Figure S1. Baseline characteristics of the whole population and a comparison of the cohorts using TE and 2D‐SWE are shown in Table 1.

**TABLE 1:** Baseline features of these 104 patients and the comparison of the cohorts using transient elastography (TE) and two‐dimensional shear wave elastography (2D‐SWE)

Characteristics	Overall (n = 104)	LSM by TE cohort (n = 102)	LSM by 2D‐SWE cohort (n = 88)
Age – y	53.4 ± 12.6	53.4 ± 12.6	53.2 ± 12.7
Sex, female, n (%)	43 (41.3)	43 (42.2)	33 (57.5)
BMI – kg/m^2^	30.9 ± 7.2	32.3 ± 7.2	31.6 ± 7.1
BMI – kg/m^2^, n (%)			
<25	8 (7.6)	8 (9.3)	8 (9.4)
25‐29.9	37 (35.5)	32 (37.2)	32 (37.6)
≥30	59 (56.7)	47 (53.5)	45 (52.9)
Diabetes mellitus, n (%)	48 (47.1)	47 (46.1)	39 (44.3)
Arterial hypertension, n (%)	54 (52.0)	52 (51.0)	45 (51.0)
Dyslipidaemia, n (%)	54 (52.5)	53 (52.0)	45 (51.0)
ALT – IU/L	75.7 ± 45	80.7 ± 57.2	84.4 ± 58.4
AST – IU/L	63.7 ± 41.1	62.6 ± 40.2	65.0 ± 41.4
AST/ALT ratio	0.9 ± 0.4	0.9 ± 0.4	0.9 ± 0.4
Bilirubin – µmol/L	10.8 ± 6.9	10.6 ± 6.8	10.8 ± 6.4
GGT – IU/L	166 ± 240	164 ± 242	175 ± 258
Cholesterol – mmol/L	4.8 ± 1.21	4.8 ± 1.2	4.8 ± 1.20
HDL – mmol/L	1.1 ± 0.3	1.1 ± 0.3	1.1 ± 0.3
Triglycerides – mmol/L	2.2 ± 1.6	2.2 ± 1.6	2.2 ± 1.7
Glucose – mmol/L	6.3 ± 2.2	6.3 ± 2.2	6.2 ± 2.1
Insulin – mU/L	31.7 ± 29.1	31.7 ± 29.6	31.9 ± 31
Platelet count – g/L	220.8 ± 70.8	220 ± 70	223.4 ± 70.6
Albumin – g/L	38.8 ± 5.0	38 ± 4.9	38.7 ± 3.5
CAP value – (dB/m)	321.0 ± 47.6	320.3 ± 47.6	316.7 ± 46.8
LSM (TE) – kPa	11.9 ± 8.0	11.9 ± 7.9	11.1 ± 5.4
LSM (2D‐SWE) – kPa	9.5 ± 4.4	9.5 ± 4.4	9.4 ± 4.3
Histology at biopsy			
Steatosis grade			
S0 (<5%)	0 (0)	0 (0)	0 (0)
S1 (5%‐33%)	27 (26.0)	27 (26.5)	19 (22.6)
S2 (34%‐66%)	31 (29.8)	29 (28.4)	29 (33.0)
S3 (>66%)	46 (44.2)	46 (45.1)	40 (45.5)
Steatosis %	55.3 ± 26.6	55.2 ± 26.8	57.3 ± 25.4
Activity			
A0	9 (8.7)	9 (8.8)	8 (9.1)
A1	9 (8.7)	9 (8.8)	7 (8.0)
A2	63 (60.6)	61 (59.8)	55 (62.5)
A3	20 (19.2)	20 (19.6)	15 (17.0)
A4	3 (2.9)	3 (2.9)	3 (3.4)
Fibrosis stage			
F0	11 (10.6)	11 (10.8)	10 (11.4)
F1	13 (12.5)	12 (11.8)	13 (14.8)
F2	35 (33.7)	35 (34.3)	29 (33.0)
F3	37 (35.6)	36 (35.4)	30 (34.1)
F4	8 (7.7)	8 (7.8)	6 (6.8)
Ballooning stage			
0	14 (13.5)	14 (13.7)	12 (13.6)
1	71 (68.3)	69 (67.6)	62 (70.5)
2	18 (17.3)	18 (17.6)	13 (14.8)
3	1 (1.0)	1 (1.0)	1 (1.1)
Lobular infiltration			
0	12 (11.5)	12 (11.8)	10 (11.4)
1	82 (78.8)	80 (78.4)	71 (80.7)
2	10 (9.6)	10 (9.8)	7 (8.0)
NAS score			
0‐2	13 (12.6)	13 (12.9)	10 (11.5)
3‐4	78 (75.7)	76 (75.2)	67 (77.0)
5‐8	12 (11.7)	12 (11.9)	10 (11.5)

Note

Data are given as mean ± standard deviation or as number of cases (percentage).

Abbreviations: ALT, alanine aminotransferase; BMI, body mass index; GGT, gamma‐glutamyl transpeptidase; HDL, high‐density lipoprotein; HOMA, homeostasis model assessment; IU, international units; kPa, kilopascal; y, years.

The M probe and XL probe were used in 38.4% and 61.5% of patients respectively. Among XL probe patients, the BMI >30 kg/m^2^ criteria was used in 92%, and the skin‐to‐capsule distance criteria ≥25 mm was used in 8%. Technical failure to measure liver stiffness occurred in none of the cases with TE and in 15 of the cases (13%) with 2D‐SWE because of the inability to obtain an adequate signal for the acquisitions. Unreliable LSM was observed in two cases (1.9%) with TE and in one case (0.9%) with 2D‐SWE. Failure or unreliable results of LSM with 2D‐SWE were associated with higher BMI (Spearman's correlation coefficient [*ρ*] = 0.32; 95%CI, 0.14‐0.49; *P* < .001), higher values of LSM by TE (*ρ* = 0.22; 95%CI, 0.03‐0.40; *P* = .02) and higher values of CAP (*ρ* = 0.21; 95%CI, 0.02‐0.39; *P* = .03).

**FIGURE 1:**
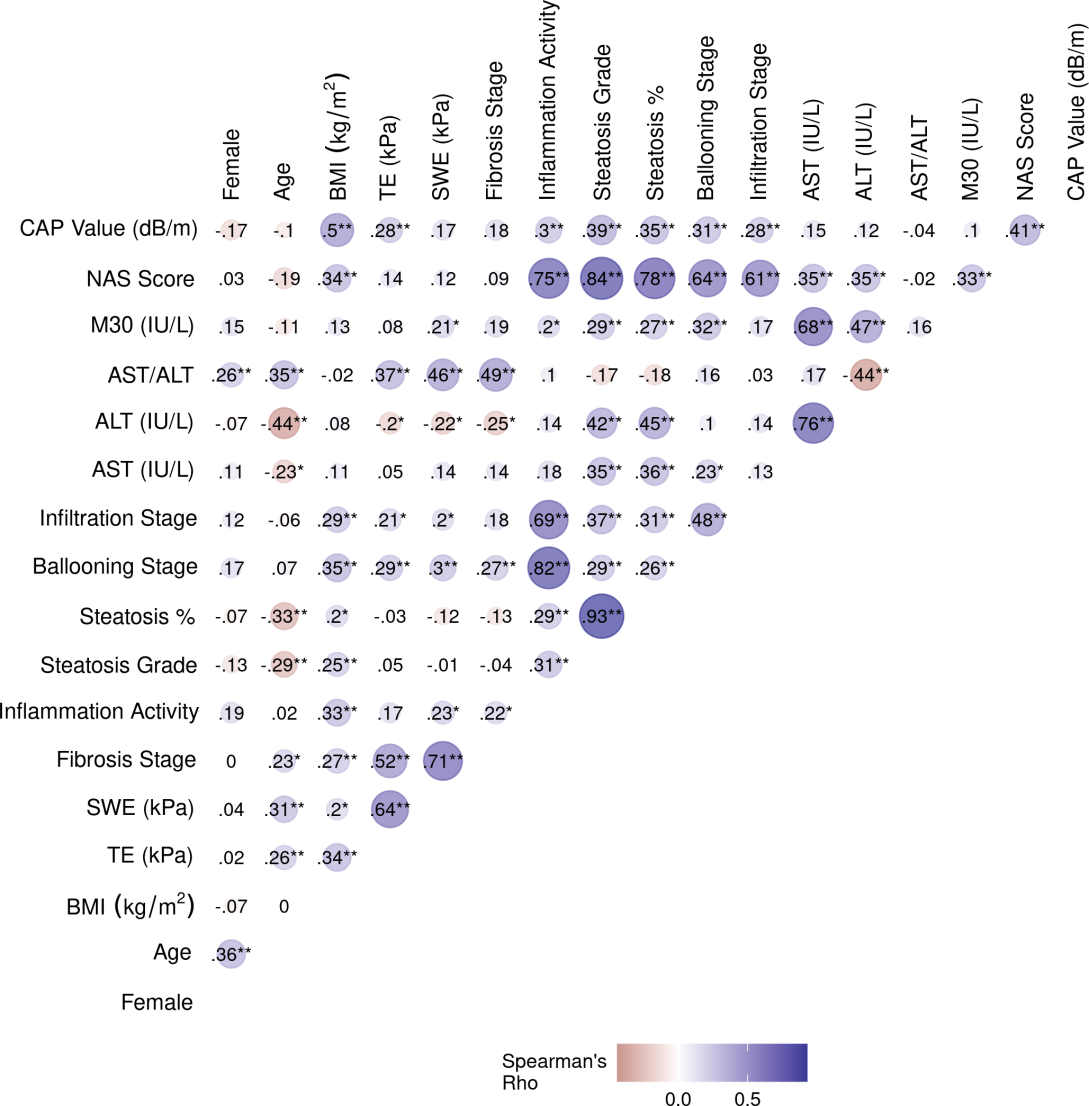
Spearman's correlations for patient characteristics, liver histology, laboratory parameters and liver stiffness by two non‐invasive tests (2D‐SWE and TE). Of note, the correlation of histological fibrosis stage with liver stiffness was stronger for 2D‐SWE than for TE. *P < .05; **P < .01

LSM by TE and 2D‐SWE was correlated with the histological fibrosis stage (Figure 1). Notably, the correlation of histological fibrosis stage with liver stiffness was stronger for 2D‐SWE (*ρ* = 0.71; 95%CI, 0.59‐0.79; *P* < .001) than for TE (*ρ* = 0.52; 95%CI, 0.37‐0.65; *P* < .001; *Z* = 2.21; *P* = .02). LSM by TE and 2D‐SWE was strongly correlated with each other (*ρ* = 0.64; 95%CI, 0.50‐0.74; *P* < .001).

Table 2 shows the diagnostic performance of LSM by TE cut‐off values for fibrosis described recently by Eddows et al for NAFLD patients,^11^ namely 8.2, 9.7 and 13.6 kPa for each fibrosis stage F ≥ F2, F ≥ F3 and F = F4 respectively. LSM by TE had good sensitivity and specificity with a good PPV (0.88) for ≥F2 and an excellent NPV (0.98) for F4. The discriminative capacity between F0‐F2 vs F3 was lower with an AUROC of 0.72 (0.63‐0.82) at a threshold of 9.7 kPa. False negative rate of LSM by TE for significant fibrosis (≥F2) was 16%. The diagnostic performance of LSM by 2D‐SWE using the cut‐off proposed for NAFLD patients^20^ is also detailed in Table 2. It showed good diagnostic performance for fibrosis stages F ≥ F2 and F = F4. The discriminative ability of LSM by 2D‐SWE was numerically higher than by TE for distinguishing F0‐F2 vs F3 with an AUROC of 0.84 (0.76‐0.92) at a threshold of 9.2 kPa.

**TABLE 2:** Diagnostic performance of LSM by TE and 2D‐SWE for each fibrosis stage

LSM by TE			
	F ≥ F2	F ≥ F3	F = F4
AUROC (95%CI)	0.76 (0.64‐0.88)	0.72 (0.63‐0.82)	0.89 (0.78‐1.00)
Cut‐off (kPa)	8.2	9.7	13.6
Se (95%CI)	0.83 (0.75‐0.89)	0.73 (0.64‐0.81)	0.87 (0.79‐0.92)
Sp (95%CI)	0.62 (0.52‐0.71)	0.53 (0.43‐0.62)	0.77 (0.68‐0.84)
PPV (95%CI)	0.88 (0.80‐0.93)	0.55 (0.46‐0.64)	0.24 (0.16‐0.33)
NPV (95%CI)	0.53 (0.44‐0.62)	0.72 (0.62‐0.79)	0.98 (0.94‐0.99)
FPR (95%CI)	0.37 (0.28‐0.47)	0.46 (0.37‐0.56)	0.22 (0.15‐0.31)
FNR (95%CI)	0.16 (0.10‐0.24)	0.26 (0.18‐0.35)	0.12 (0.07‐0.20)
LSM by 2D‐SWE			
AUROC (95%CI)	0.83 (0.72‐0.93)	0.84 (0.76‐0.92)	0.94 (0.89‐0.99)
Cut‐off (kPa)	7.1	9.2	13
Se (95%CI)	0.86 (0.79‐0.91)	0.65 (0.64‐0.81)	0.83 (0.75‐0.89)
Sp (95%CI)	0.73 (0.64‐0.81)	0.86 (0.78‐0.91)	0.87 (0.80‐0.92)
PPV (95%CI)	0.90 (0.83‐0.94)	0.78 (0.69‐0.84)	0.33 (0.25‐0.42)
NPV (95%CI)	0.65 (0.55‐0.73)	0.77 (0.62‐0.79)	0.98 (0.94‐0.99)
FPR (95%CI)	0.26 (0.18‐0.35)	0.13 (0.08‐0.21)	0.33 (0.25‐0.42)
FNR (95%CI)	0.13 (0.08‐0.21)	0.34 (0.25‐0.43)	0.16 (0.10‐0.24)

Abbreviations: FNR, false negative rate; FPR, false positive rate; NPV, negative predictive value; PPV, positive predictive value; Se, sensitivity; Sp, specificity.

The AUROC and false positive rate for LSM by TE and 2D‐SWE for diagnosing histological fibrosis stage according to the histological inflammatory activity stage are summarized in Table S2.

### 3.2 Potential confounders of the association between fibrosis and liver stiffness measured by TE and 2D‐SWE

We calculated a baseline linear regression model of the fibrosis stage on the LSM. To assess whether the histological steatosis, inflammatory activity, NAS score and laboratory parameters would influence the association between fibrosis and LSM by TE or 2D‐SWE, we compared all further models against this baseline model.

#### 3.2.1 Influence of inflammation and steatosis on LSM by TE

**FIGURE 2:**
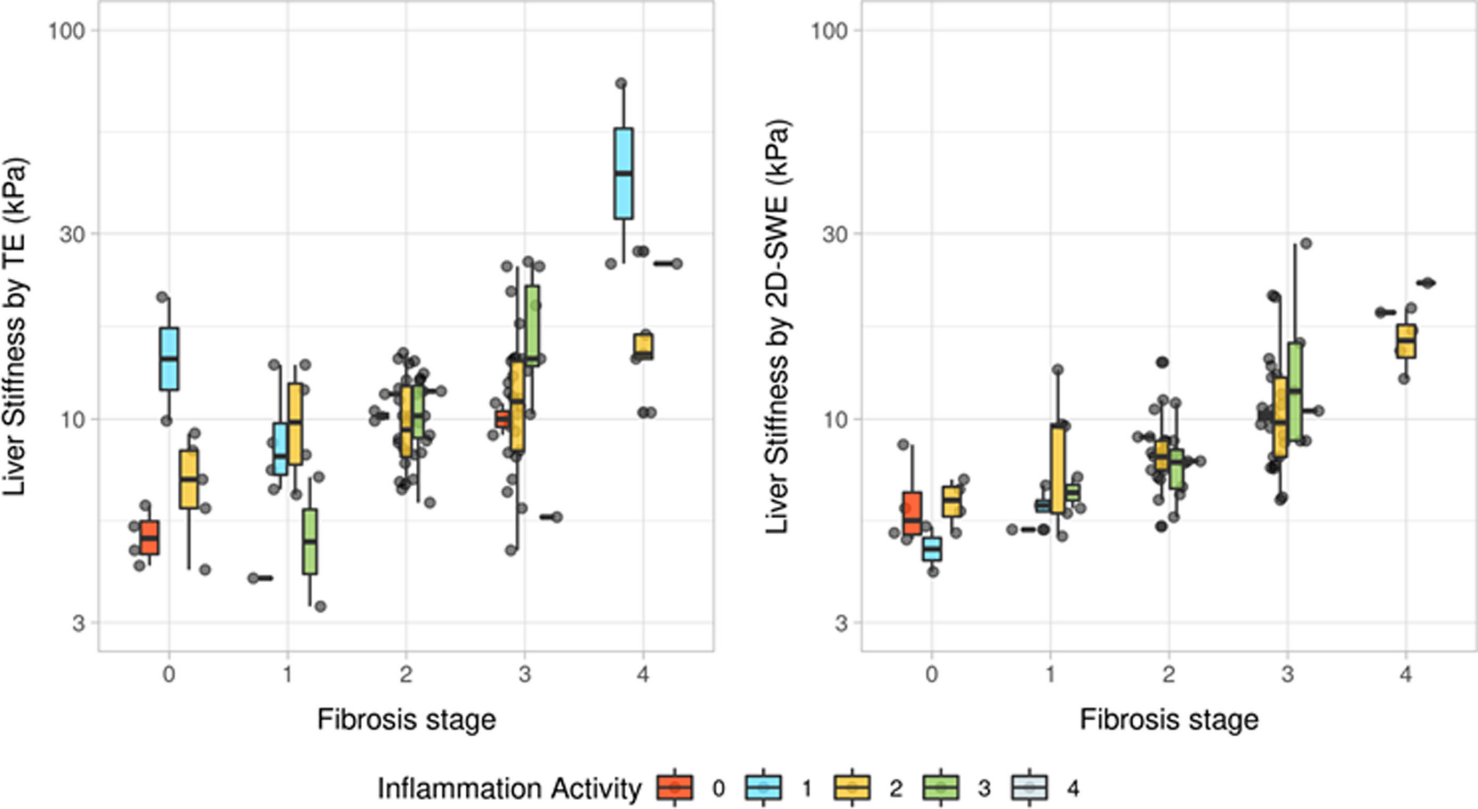
Boxplot of liver stiffness measurement (LSM) in patients with non‐alcoholic fatty liver disease using transient elastography (TE) and two‐dimensional shear wave elastography (2D‐SWE) by fibrosis stage and inflammatory activity. When using TE, the association of histological fibrosis and LSM changes according to the level of inflammatory activity

The fibrosis stage on histology explained 35% of the variance of LSM by TE (*P* < .001). The variance explained by the model did not increase significantly compared to the baseline model after including the main effect of steatosis percentage and the interaction of steatosis percentage and fibrosis stage ($R_{\text{cha}}^{2}$ = .04; *P* = 1) (Table 3). When including inflammatory activity and the interaction between inflammatory activity and histological fibrosis stage, the model explained 25% more variance in LSM by TE (*P* < .01). After controlling for fibrosis, age, sex and BMI, both the main effect of inflammatory activity (*P* < .001) and the interaction effect between inflammatory activity and fibrosis (*P* = .01) remained significantly associated with LSM by TE, and independently explained 11% and 13% of variance in LSM by TE respectively (Table 4). This indicates that the strength of the association between fibrosis on histology and LSM by TE changes according to the severity of the inflammatory activity. This effect is visualized in Figure 2. Regression results for the individual combinations of fibrosis stage and grade of inflammatory activity are provided in Tables S3 and S4.

**TABLE 3:** Model comparisons against the baseline model with fibrosis stage on the liver stiffness measurement (LSM) by transient elastography (TE) and by two‐dimensional shear wave elastography (2D‐SWE)

Outcome	LSM by TE			LSM by 2D‐SWE		
Model	*F* (*df*)	$R_{\text{cha}}^{2}$	*P*‐value	*F* (*df*)	$R_{\text{cha}}^{2}$	*P*‐value
Baseline: Fibrosis stage	12.81 (4, 85)	.35	<.001	22.15 (4, 81)	.52	<.001
+Steatosis (%), interaction	1.25 (5, 88)	.04	1	0.15 (5, 74)	.00	1
+Inflammatory activity, interaction	3.56 (20, 81)	.25	<.01	0.78 (20, 68)	.06	1

Note

*P*‐values Holm‐adjusted for multiple comparisons. *F* is the ratio of explained variance between the baseline model and those with added predictors.

**TABLE 4:** Logistic regression models with liver stiffness measurement (LSM) by transient elastography (TE) as the dependent variable

Predictor (*df*)	Model 1			Model 2			Model 3		
	*F*	η^2^	*P*‐value	*F*	η^2^	*P*‐value	*F*	η^2^	*P*‐value
Fibrosis stage (4)	12.81	0.37	<.001	17.54	0.35	<.001	18.36	0.35	<.001
Inflammatory activity (4)				5.76	0.11	<.001	6.04	0.11	<.001
Inflammatory activity				2.68	0.13	<.01	2.70	0.13	<.01
Fibrosis score (10)									
Age (1)							4.32	0.02	.03
BMI (1)							3.69	0.02	.05
Gender (1)							0.95	0.00	.33
Residual *df*	95			81			77		
*R* ^2^ (95%CI)	.35 (.20; .49)			.59 (.23; .70)			.63 (.50; .73)		

Note

All models were fit to *m* = 20 multiply imputed data sets based on 102 patients with reliable LSM by TE.

Furthermore, we tested whether the variance explained by the fibrosis stage would be increased by including individual inflammatory activity components (ballooning and lobular Infiltration), NAS score, M30, ratio of AST/ALT, CAP or their interaction. None of these models showed a significant increase in the explained variance (Table S5), suggesting that the strength of the association between liver fibrosis and LSM is mostly modified by the overall severity of inflammation.

#### 3.2.2 Influence of inflammation and steatosis on LSM by 2D‐SWE

The same analysis was applied to LSM by 2D‐SWE as the dependent variable. In the baseline model, fibrosis stage on histology explained 52% in variance of LSM by 2D‐SWE (*P* < .001). Including steatosis percentage and its interaction with fibrosis stage into the model did not significantly improve the model ($R_{\text{cha}}^{2}$ = .00; *P* = 1). Similarly, including inflammatory activity and its interaction with fibrosis stage did not explain significantly more variance than the baseline model ($R_{\text{cha}}^{2}$ = .06; *P* = 1) (Table 3). We tested the same additional models as for LSM by 2D‐SWE. None of these models explained significantly more variance in the dependent variable, indicating that none of the variables altered the effect of the fibrosis stage on LSM by 2D‐SWE (Table S5).

### 3.3 Sensitivity analysis in patients with reliable LSM using both TE and 2D‐SWE methods

The analysis of the 86 patients with reliable LSM on both TE and 2D‐SWE showed similar results. Inflammatory activity and its interaction with fibrosis stage explained 27% in the variance of LSM by TE (*P* < .01). Fibrosis stage, inflammatory activity and its interaction with liver fibrosis remained significant when controlling for age, BMI and sex. Similar to the larger data set, none of the models in which LSM by 2D‐SWE was tested as the dependent variable improved the baseline model significantly. Table S6 contains the model comparisons for this sensitivity analysis.

## 4 DISCUSSION

In this prospective cohort study, we found that inflammatory activity assessed on histology significantly affects liver stiffness measured by TE, but not liver stiffness measured by 2D‐SWE in patients with NAFLD. The severity of steatosis on histology did not affect the association between LSM and fibrosis by any of the two elastography methods used. In our cohort, LSM by 2D‐SWE reflects liver fibrosis more accurately than LSM by TE in NAFLD patients. However, LSM failures were more frequent using 2D‐SWE than TE owing to the technical limitations – mostly because of obesity likely impeding an efficient transmission of the ultrasound waves to the liver.

It has been reported that 2D‐SWE has a higher rate of failure in patients with high BMI.^21^ This is likely the reason why we experienced a relatively high rate of 2D‐SWE failures in our cohort composed mostly of overweight and obese patients. Nevertheless, the 13% failure rate of LSM by 2D‐SWE is in agreement with that reported in the study by Cassinotto et al.^15^ In addition, in our study, we found a lower unreliable/failure rate of LSM by TE than previously reported (1.9% vs 14%), likely because the XL probe was available for patients with obesity and/or skin‐to‐capsule distance ≥25 mm. Our findings are in keeping with studies using XL probe, which have reported a high rate of LSM success with TE (97% of patients could be evaluated successfully).^11^, ^22^

The diagnostic performance of LSM by TE and by 2D‐SWE for fibrosis stages in this study is also in line with data from large cohorts.^11^, ^20^ Our results are in agreement with the previous data by Cassinotto et al regarding a lack of influence of steatosis and inflammatory activity on LSM by 2D‐SWE, and confirm that LSM by 2D‐SWE is accurate in predicting fibrosis in NAFLD patients. Consistent with previous reports,^10^, ^11^, ^12^ we found that steatosis did not affect LSM by TE. We could not confirm the association between steatosis and liver stiffness supported by the study by Petta et al,^13^ which, however, used exclusively the M probe, suggesting a potential technical error as a result of inappropriate elastography frequency. Our results are similar to those shown in recent cohorts,^11^, ^12^ in which M and XL probes were used. For example, Wong et al^12^ used M and XL probes in patients with BMI <30 and ≥30 kg/m^2^, respectively, similar to our criteria, showing that hepatic steatosis did not affect the performance of LSM by TE. Therefore, studies using only the M probe are likely to give an incorrect interpretation in many patients with NAFLD.

The liver is a visco‐elastic structure. Most of the ultrasound elastography methods implement a simple linear elastic model that quantifies the tissue elasticity but not the liver viscosity, which has been associated with necro‐inflammatory activity.^23^ Recent experimental^23^ and clinical^24^ studies showed that LSM assessment using 2D‐SWE method reflects elasticity but does not reflect viscosity. Our results are in line with this observation, and suggest that while liver stiffness measured by TE allows an overall assessment of visco‐elastic properties of the liver tissue, it is more susceptible to the confounding effect of inflammation as compared to methods purely designed to reflect the liver elastic properties such as 2D‐SWE. New techniques known as shear wave dispersion imaging^24^ and shave wave spectrometry^25^ have been proposed to measure viscosity by analysing dispersion properties of the wave propagation in the tissue.^26^ To what extent, however, viscosity and elasticity can truly be differentiated is matter of debate, since they are correlated.^25^

Interestingly, Petta et al^13^ reported that inflammation was an independent predictor of a higher LSM by TE (*P* < .001). Our study further expands these previous findings supporting that not only inflammation, but also the interaction between inflammatory activity and fibrosis affects the reliability of LSM by TE on fibrosis assessment in NAFLD. This may be interpreted as a limitation of TE for fibrosis assessment in NAFLD. The combination of unrelated non‐invasive methods to predict fibrosis has emerged to overcome the limitation of individual tests. In fact, in NAFLD patients, recent studies found that the combination or sequential use of a second elastography technique or serum‐based tests improves the predictive ability for fibrosis.^27^, ^28^

On the other hand, non‐invasive tests identifying inflammation in NAFLD are lacking. Since inflammation seems to increase LSM using TE but not using 2D‐SWE, one could hypothesize that combining both TE and 2D‐SWE could help identify those patients in whom inflammation is present, namely those showing a higher LSM by TE compared to 2D‐SWE. This would have particular relevance when considering that the NAFLD field is seeking a non‐invasive test that identifies patients with fibrosis and clinically active NASH suitable for clinical trials on novel therapies, and could be object of the future studies.

This study has limitations. Some of them are inherent to liver biopsies, such as sampling error and interobserver variability.^8^ We strived to overcome this by following the specific quality criteria (length of sample; interpretation from an expert liver pathologist) and using predefined histological scoring systems. Another limitation was related to the heterogeneous distribution of cases in the different subgroups of histological fibrosis and steatosis or inflammation, which resulted in a low number of cases in some subgroups. Therefore, to avoid misleading results, we refrained from performing subgroup analysis of these combinations and instead focused on the global effects of the predictors. Not all patients had valid LSM using both elastography techniques, leading to a possible bias in the interpretation of the results; however, sensitivity analysis on patients with valid measurements in both techniques confirmed the findings of the overall cohort.

There are several strengths of this study. The population is a well‐characterized prospective cohort in whom the use of the optimal TE probe (either M or XL) was used to characterize NAFLD. Moreover, the statistical analysis is done using a principled approach where predictors are tested against a meaningful baseline model rather than a simple null model. This has the advantage of adding explanatory weight to observed phenomena, while fully utilizing the limited available data.

In summary, we found that in NAFLD patients, the association between fibrosis and liver stiffness using 2D‐SWE is not altered by steatosis and inflammatory activity. Furthermore, LSM by 2D‐SWE was better correlated with the fibrosis stage. On the other hand, liver fibrosis assessment by LSM using TE was also not affected by steatosis, but it was affected by the histological degree of inflammatory activity and its interaction with fibrosis, which would result in an over interpretation of liver fibrosis. According to our data, we conclude that liver stiffness by 2D‐SWE appears to reflect fibrosis more accurately in patients with NAFLD when technically feasible.

## CONFLICT OF INTEREST

The authors do not have any disclosures to report.

## AUTHOR CONTRIBUTIONS

YPM: data collection, analysis of the data and drafting the manuscript; SGR, MGD and GM: data collection and manuscript revision; NFL and MM: manuscript revision; JS: statistical analysis of the data and manuscript revision; JFD: critical revision of the manuscript for important intellectual content; AB: study conception, supervision of the study, manuscript revision for important intellectual content. All authors have commented on the manuscript and approved the final version.

## Supporting information

Supplementary Material

## Data Availability

The data that support the findings of this study are available from the corresponding author upon reasonable request.
