# Supplementary material for: Inflammatory activity affects the accuracy of liver stiffness measurement by transient elastography but not by two‐dimensional shear wave elastography in non‐alcoholic fatty liver disease
Source: Liver Int. 2021 Dec 3;42(1):102–11. doi: 10.1111/liv.15116 (PMC9299715; doi:10.1111/liv.15116)
Supplement: Supplementary file 1 — Supplementary Material [file LIV-42-102-s001.docx]

**Inflammatory activity affects the accuracy of liver stiffness measurement by transient elastography (TE), but not by two-dimensional shear wave elastography (2D-SWE) in nonalcoholic fatty liver disease**

**Authors**: Yuly P. Mendoza^1,2,3^, Susana G. Rodrigues^1,2^, Maria G. Delgado^1,2^, Giuseppe Murgia^1^, Naomi F. Lange^1,2^, Jonas Schropp^1,5,6^, Matteo Montani^4^, Jean-François Dufour^1,2^, Annalisa Berzigotti^1,2^

Table of contents

Methods………………………………………………………………………………………2

Results…………….......................................................................................................2

Supplementary Figure 1..………………………………………………………………..…3

Supplementary Tables ..……………………………………………………………………4

**Methods**

*Statistical analysis*

We report the 95% confidence intervals of the mean difference, mean difference in ranks and mean difference in proportions, respectively, and indicate significance.

Multivariate Imputation by Chained Equations (MICE) was used to handle missing values on all predictor variables. Several laboratory parameters - albumin, bilirubin, gGT, cholesterol, HDL cholesterol, triglycerides, HbA1c, and platelet count - were used as auxiliary variables, but not further examined in the context of the present study. NAS score was calculated after imputation of steatosis level and inflammation activity, AST/ALT ratio after imputation of AST and ALT.

The comparison of models allows evaluation of the global effect of adding the covariate rather than differences between the individual levels of (predominantly categorical) predictors.

**Results**

Because the distribution of cases in the different subgroups of histologic fibrosis and inflammation is very heterogeneous, resulting in an insufficient number of cases in some combinations, we refrain from closely interpreting regression weights for these combinations and rather focus on the global effects of predictors. However, more detailed results of the regression models which significantly improved upon the baseline model can be seen in supplementary table 1.

**Supplementary Figure 1.** Study flow chart. Of 137 patients enrolled, 104 were eligible, 102 had reliable liver stiffness measurement (LSM) by transient elastography (TE) and 82 had reliable LSM by two two-dimensional shear wave elastography (2D-SWE).


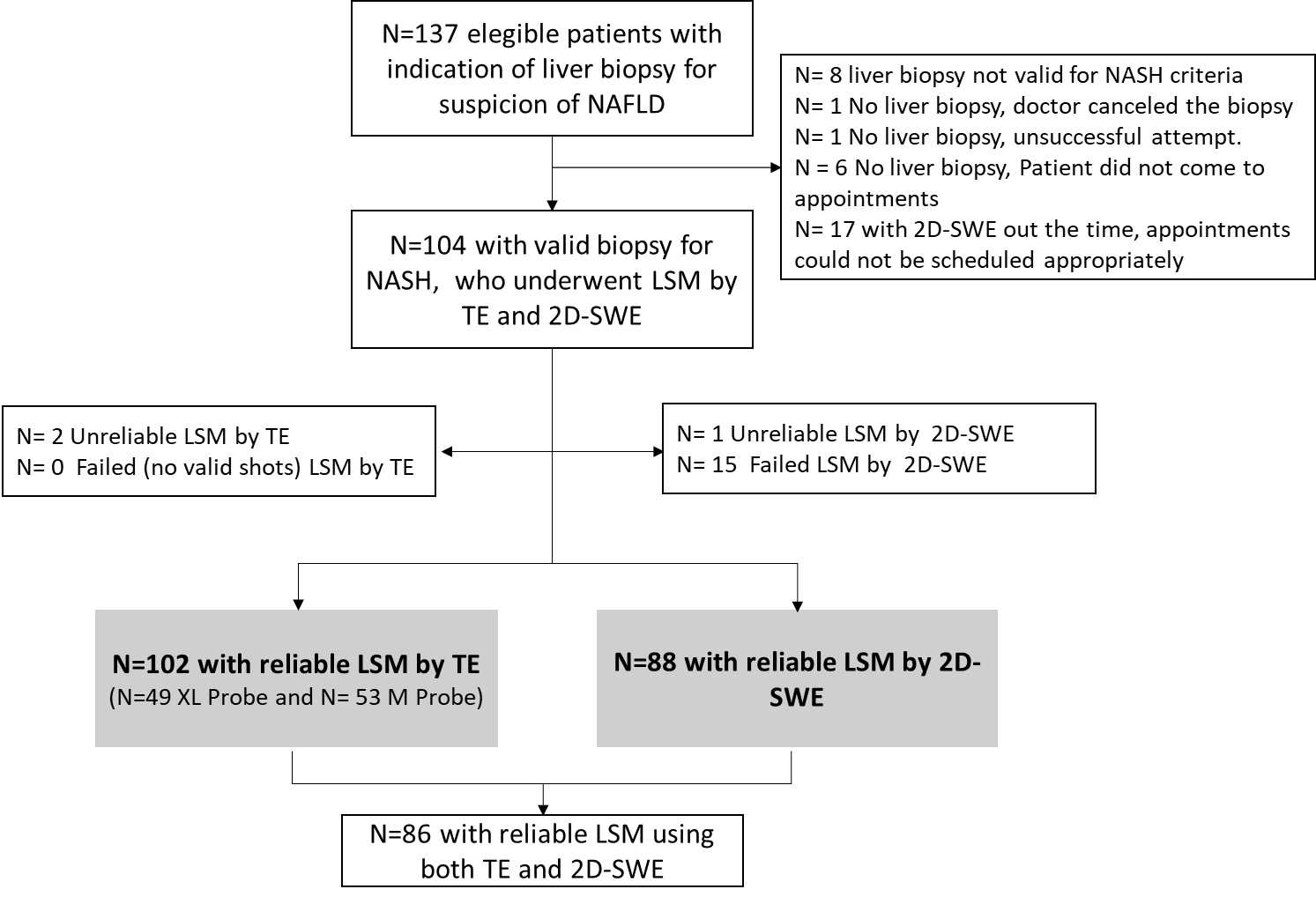


**Supplementary Table 1.** Differences in characteristics between the 88 patients with reliable LSM by 2D-SWE and the 16 patients without reliable LSM by 2D-SWE

| **Characteristics** | **Patients with Reliable LSM by 2D-SWE (n=88)** | **Patients without Reliable LSM by 2D-SWE (n=16)** | ***P*-value** |
| --- | --- | --- | --- |
| Age – y | 53.2 ± 12.7 | 59.4±12.6 | 0.048 |
| BMI – kg/m^2^ | 31.6 ± 7.1 | 36.1 ± 7.2 | 0.018 |
| Diabetes Mellitus, n (%) | 39 (44.3) | 48 (47.1) | 0.775 |
| Arterial hypertension, n (%) | 45 (51.0) | 54 (52.0) | 0.069 |
| Dyslipidemia, n (%) | 45 (51,0) | 54 (52.5) | 0.480 |
| ALT– IU/L | 84.4 ± 58.4 | 62.5± 45 | 0.139 |
| AST - IU/L | 65.0±41.4 | 58.0±41.1 | 0.598 |
| AST/ALT ratio | 0.9±0.4 | 0.9±0.4 | 0.381 |
| Bilirubin - µmol/L | 10.8 ± 6.4 | 10.7 ± 6.9 | 0.979 |
| Cholesterol – mmol/L | 4.8 ± 1.20 | 4.9 ± 1.21 | 0.685 |
| HDL – mmol/L | 1.1 ± 0.3 | 1.15 ± 0.3 | 0.684 |
| Triglycerides – mmol/L | 2.2 ± 1.7 | 3.3 ± 1.6 | 0.523 |
| Glucose – mmol/L | 6.2 ± 2.1 | 7.1 ± 2.2 | 0.132 |
| Insulin – mU/L | 31.9 ± 31 | 29.5 ± 29.1 | 0.806 |
| Platelet count – g/L | 223.4 ± 70.6 | 205.8 ± 70.8 | 0.397 |
| Albumin – g/L | 38.7 ±3.5 | 33.6 ± 5.0 | 0.072 |
| CAP Value - (dB/m) | 316.7 ± 46.8 | 344.0 ± 47.6 | 0.058 |
| LSM (TE) - kPa | 11.1 ± 5.4 | 16.5 ± 8.0 | 0.226 |
| **Histology at biopsy**  Steatosis grade  S0 (<5%)  S1 (5%-33%)  S2 (34%-66%)  S3 (>66%) | 0 (0)  19 (22.6)  29 (33.0)  40 (45.5) | 0 (0)  8 (50)  2 (12.5)  6 (37.5) | 0.059 |
| Activity  A0  A1  A2  A3  A4 | 8 (9.1)  7 (8.0)  55 (62.5)  15 (17.0)  3 (3.4) | 1 (6.3)  2 (12.5)  8 (50)  5 (31.3)  0 (0) | 0.580 |
| Fibrosis stage  F0  F1  F2  F3  F4 | 10 (11.4)  13 (14.8)  29 (33.0)  30 (34.1)  6 (6.8) | 0 (0)  1 (6.3)  6 (37.5)  7 (43.7)  2 (12.5) | 0.198 |

**Supplementary table 2.** Areas under the receiver operating characteristic curve (AUROC) and False-positivity rate (with 95% confidence interval) for the diagnostic accuracy of LSM by TE and 2D-SWE for the diagnosis of histologic fibrosis stage according to the histological inflammatory activity stage.

| **AUROC of LSM by TE** | | | |
| --- | --- | --- | --- |
| **Inflammatory activity** | **F ≥F2** | **F≥F3** | **F=F4** |
| 0 | 0.80 (0.46-1.00) | 0.66 (0.22-1.00) | No cases |
| 1 | 0.80 (0.47-1.00) | 1.00 (1.00-1.00) | 1.00 (1.00-1.00) |
| 2 | 0.74 (0.56-0.91) | 0.71 (0.59-0.84) | 0.83 (0.67-0.99) |
| 3 | 0.97 (0.89 -1.00) | 0.94 (0.83-1.00) | No cases |
| 4 | No cases | 0.50 (NA-NA) | 1.00 (NA-NA) |
| **AUROC of LSM by 2D-SWE** | | | |
| 0 | 0.86 (0.56-1.00) | 0.86 (0.56-1.00) | No cases |
| 1 | 0.79 (0.60-1.00) | 1.00 (NA-NA) | 1.00 (NA-NA) |
| 2 | 0.79 (0.61-0.97) | 0.78 (0.67-0.90) | 0.91 (0.82-1.00) |
| 3 | 0.84 (0.61-1.00) | 0.94 (0.82-1.00) | No cases |
| 4 | No cases | 1.00 (NA-NA) | 1.00 (NA-NA) |
| **False-positivity rate of LSM by TE** | | | |
| **Inflammatory activity** | **F ≥F2** | **F≥F3** | **F=F4** |
| 0 | 0.20 (0.53-0.52) | 0.33 (0.12-0.64) | No cases |
| 1 | 0.60 (0.30-0.83) | 0.57 (0.27-0.82) | 1.00 (1.00-1.00) |
| 2 | 0.41 (0.30-0.53) | 0.41 (0.30-0.32) | 0.83 (0.67-0.99) |
| 3 | 0.00 | 0.53 (0.00-0.73) | No cases |
| 4 | No cases | 1.00 | 1.00 (NA-NA) |
| **False-positivity rate of LSM by 2D-SWE** | | | |
| 0 | 0.40 (0.16-0.69) | 0.00 | No cases |
| 1 | 0.00 | 0.00 | 0.00 |
| 2 | 0.27 (0.17-0.39) | 0.20 (0.12-0.32) | 0.15 (0.82-0.26) |
| 3 | 0.50 (0.29-0.70) | 0.1 (0.02-0.30) | No cases |
| 4 | No cases | 0.00 | 0.00 |

**Supplementary table 3.** Regression results of model 2 using ln LSM (kPa) as the criterion

| Predictor | *b*, 95% CI [LL, UL] | *p* | *r_part_*, 95% CI [LL, UL] |
| --- | --- | --- | --- |
| (Intercept) | 1.61, [1.28, 1.94] | <.001 |  |
| F1 | -0.24, [-0.98, 0.50] | .517 | -0.07, [-0.28, 0.14] |
| F2 | 0.72, [0.15, 1.29] | .016 | 0.26, [ 0.05, 0.44] |
| F3 | 0.70, [0.13, 1.27] | .019 | 0.26, [ 0.04, 0.44] |
| F4 | 2.20, [1.11, 3.29] | <.001 | 0.40, [ 0.21, 0.55] |
| I1 | 1.05, [0.48, 1.62] | .001 | 0.37, [ 0.17, 0.53] |
| I2 | 0.29, [-0.15, 0.73] | .204 | 0.14, [-0.08, 0.34] |
| I3 | 0.50, [-0.03, 1.03] | .065 | 0.20, [-0.01, 0.39] |
| I4 | -0.58, [-1.39, 0.23] | .162 | -0.16, [-0.35, 0.06] |
| Int F1*I1 | -0.25, [-1.18, 0.68] | .606 | -0.06, [-0.27, 0.16] |
| Int F2*I1 | -0.92, [-1.91, 0.07] | .070 | -0.20, [-0.39, 0.02] |
| Int F3*I1 | - | - | - |
| Int F4*I1 | -1.10, [-2.37, 0.17] | .094 | -0.18, [-0.38, 0.03] |
| Int F1*I2 | 0.54, [-0.30, 1.38] | .210 | 0.14, [-0.08, 0.34] |
| Int F2*I2 | -0.34, [-1.00, 0.32] | .314 | -0.11, [-0.31, 0.11] |
| Int F3*I2 | -0.19, [-0.84, 0.46] | .566 | -0.06, [-0.27, 0.15] |
| Int F4*I2 | -1.33, [-2.50, -0.16] | .028 | -0.24, [-0.42, -0.03] |
| Int F1*I3 | -0.29, [-1.25, 0.67] | .560 | -0.06, [-0.27, 0.15] |
| Int F2*I3 | -0.51, [-1.24, 0.22] | .175 | -0.15, [-0.35, 0.07] |
| Int F3*I3 | - | - | - |
| Int F4*I3 | - | - | - |
| Int F1*I4 | - | - | - |
| Int F2*I4 | 0.73, [-0.41, 1.87] | .215 | 0.14, [-0.08, 0.34] |
| Int F3*I4 | - | - | - |
| Int F4*I4 | - | - | - |

F0 to F4 = Fibrosis stage. I0 to I4 = Inflammation level. Int = interaction. A significant *b*-weight indicates the partial correlation is also significant. *b* represents unstandardized regression weights. *r_part_* represents the partial correlation. *LL* and *UL* indicate the lower and upper limits of a confidence interval, respectively.  *R^2^* = .59**. - represents regression weights that could not be estimated due to insufficient cases in the grouping combination.

**Supplementary table 4.** Regression results of model 3 using ln LSM (kPa) as the criterion

| Predictor | *b*, 95% CI [LL, UL] | *p* | *r_part_*, 95% CI [LL, UL] |
| --- | --- | --- | --- |
| (Intercept) | 1.05, [0.52, 1.58] | <0.001 |  |
| F1 | -0.08, [-0.82, 0.66] | 0.839 | -0.02, [-0.24, 0.2] |
| F2 | 0.65, [0.09, 1.21] | 0.027 | 0.25, [0.03, 0.43] |
| F3 | 0.58, [0, 1.16] | 0.051 | 0.22, [0, 0.41] |
| F4 | 1.98, [0.88, 3.08] | 0.001 | 0.37, [0.17, 0.53] |
| I1 | 1, [0.44, 1.56] | 0.001 | 0.37, [0.16, 0.53] |
| I2 | 0.27, [-0.17, 0.71] | 0.232 | 0.14, [-0.09, 0.34] |
| I3 | 0.52, [-0.03, 1.07] | 0.065 | 0.21, [-0.01, 0.4] |
| I4 | -0.4, [-1.22, 0.42] | 0.335 | -0.11, [-0.32, 0.11] |
| Age | 0, [0, 0] | 0.084 | 0.2, [-0.03, 0.39] |
| Bmi (Kg/m2) | 0.01, [0, 0.02] | 0.040 | 0.23, [0.01, 0.42] |
| Gender (m=0; f=1) | -0.07, [-0.23, 0.09] | 0.376 | -0.1, [-0.31, 0.12] |
| Int F1*I1 | -0.33, [-1.25, 0.59] | 0.488 | -0.08, [-0.29, 0.14] |
| Int F2*I1 | -0.89, [-1.86, 0.08] | 0.076 | -0.2, [-0.39, 0.02] |
| Int F3*I1 | - | - | - |
| Int F4*I1 | -0.95, [-2.22, 0.32] | 0.147 | -0.16, [-0.36, 0.06] |
| Int F1*I2 | 0.37, [-0.49, 1.23] | 0.405 | 0.09, [-0.13, 0.3] |
| Int F2*I2 | -0.32, [-0.97, 0.33] | 0.330 | -0.11, [-0.32, 0.11] |
| Int F3*I2 | -0.18, [-0.83, 0.47] | 0.593 | -0.06, [-0.27, 0.16] |
| Int F4*I2 | -1.24, [-2.4, -0.08] | 0.039 | -0.23, [-0.42, -0.01] |
| Int F1*I3 | -0.58, [-1.57, 0.41] | 0.258 | -0.13, [-0.33, 0.09] |
| Int F2*I3 | -0.54, [-1.26, 0.18] | 0.146 | -0.16, [-0.36, 0.06] |
| Int F3*I3 | - | - | - |
| Int F4*I3 | - | - | - |
| Int F1*I4 | - | - | - |
| Int F2*I4 | 0.58, [-0.54, 1.7] | 0.316 | 0.11, [-0.11, 0.32] |
| Int F3*I4 | - | - | - |
| Int F4*I4 | - | - | - |

F0 to F4 = Fibrosis stage. I0 to I4 = Inflammation level. Int = interaction. A significant *b*-weight indicates the partial correlation is also significant. *b* represents unstandardized regression weights. *r_part_* represents the partial correlation. *LL* and *UL* indicate the lower and upper limits of a confidence interval, respectively.  *R^2^* = .63**. - represents regression weights that could not be estimated due to insufficient cases in the grouping combination.

**Supplementary table 5**. Model comparisons against the baseline model with fibrosis stage on the natural log (ln) of LSM by TE and LSM by 2D-SWE

| Outcome | ln LSM by TE (kPa) | | | ln LSM by 2D-SWE (kPa) | | |
| --- | --- | --- | --- | --- | --- | --- |
| Model | *F (df)* | *R^2^_cha_* | p-value | *F (df)* | *R^2^_cha_* | p-value |
| Baseline: Fibrosis stage | 12.81 (4, 85) | 0.35 | < 0.001 | 22.15 (4, 81) | 0.52 | < 0.001 |
| + Steatosis (%), Interaction | 1.25 (5, 88) | 0.04 | 1 | 0.15 (5, 74) | 0.00 | 1 |
| + Inflammatory activity, Interaction | 3.56 (20, 81) | 0.25 | <0.01 | 0.78 (20, 68) | 0.06 | 1 |
| + AST/ALT, Interaction | 3.22 (5, 82) | 0.13 | 0.09 | 1.04 (5, 69) | 0.04 | 1 |
| + Ballooning, Interaction | 2.07 (16, 86) | 0.07 | 1 | 1.39 (16, 71) | 0.07 | 1 |
| + Lobular Inflamma- tion, interaction | 1.37 (15, 86) | 0.12 | 0.10 | 0.53 (15. 73) | 0.03 | 1 |
| + NAS, Interaction | 1.28 (5, 89) | 0.04 | 1 | 0.75 (5, 75) | 0.02 | 1 |
| + M30, Interaction | 0.13 (5, 70) | 0.11 | 1 | 0.69 (5, 60) | 0.03 | 1 |
| + CAP, Interaction | 2.01 (5, 92) | 0.06 | 0.42 | 1.00 (5, 78) | 0.03 | 1 |

P-values Holm-adjusted for multiple comparisons. F is the ratio of explained variance between the baseline model and those with added predictors.

Abbreviation: ln, natural Log

**Supplementary table 6**. Model comparisons against the baseline model with fibrosis stage on the natural log of LSM by TE and LSM by 2D-SWE in patients with valid measurements for both LSM by TE and 2D-SWE.

| Outcome | ln LSM by TE (kPa) | | | ln LSM by 2D-SWE (kPa) | | |
| --- | --- | --- | --- | --- | --- | --- |
| Model | *F (df)* | *R^2^_cha_* | p-value | *F (df)* | *R^2^_cha_* | p-value |
| Baseline: Fibrosis stage | 7.89 (4, 79) | 0.28 | <0.001 | 22.29 (4, 79) | 0.52 | < 0.001 |
| + Steatosis (%), Interaction | 0.37 (5, 72) | 0.01 | 1 | 0.17 (5, 72) | 0.00 | 1 |
| + Inflammation activity, Interaction | 3.13 (20, 66) | 0.27 | 0.002 | 0.73 (20, 66) | 0.06 | 1 |
| + AST/ALT, Interaction | 1.65 (5, 67) | 0.09 | 0.91 | 0.74 (5, 67) | 0.03 | 1 |
| + Ballooning, Interaction | 1.43 (16, 70) | 0.11 | 0.91 | 1.41 (16, 70) | 0.07 | 1 |
| + Infiltration, Interaction | 0.78 (15, 71) | 0.06 | 1 | 0.55 (15, 71) | 0.03 | 1 |
| + NAS, Interaction | 0.39 (5, 73) | 0.01 | 1 | 0.61 (5, 73) | 0.01 | 1 |
| + M30, Interaction | 0.26 (5, 58) | 0.09 | 1 | 0.27 (5, 58) | 0.02 | 1 |
| + CAP, Interaction | 0.13 (5, 76) | 0.08 | 0.86 | 0.48 (5, 76) | 0.03 | 1 |

P-values Holm-adjusted for multiple comparisons.

Abbreviation: ln, natural Log
